# Chimeric RXFP1 and RXFP2 Receptors Highlight the Similar Mechanism of Activation Utilizing Their N-Terminal Low-Density Lipoprotein Class A Modules

**DOI:** 10.3389/fendo.2013.00171

**Published:** 2013-11-11

**Authors:** Shoni Bruell, Roy C. K. Kong, Emma J. Petrie, Brad Hoare, John D. Wade, Daniel J. Scott, Paul R. Gooley, Ross A. D. Bathgate

**Affiliations:** ^1^Florey Department of Neuroscience and Mental Health, Florey Institute of Neuroscience and Mental Health, Melbourne, VIC, Australia; ^2^Department of Biochemistry and Molecular Biology, Melbourne, VIC, Australia; ^3^Bio21 Molecular Science and Biotechnology Institute, Melbourne, VIC, Australia; ^4^School of Chemistry, The University of Melbourne, Melbourne, VIC, Australia

**Keywords:** GPCR, signaling, relaxin, INSL3, RXFP1, RXFP2, insulin-like peptides

## Abstract

Relaxin family peptide (RXFP) receptors 1 and 2 are unique G-protein coupled receptors in that they contain an N-terminal low-density lipoprotein type A (LDLa) module which is necessary for receptor activation. The current hypothesis suggests that upon ligand binding the LDLa module interacts with the transmembrane (TM) domain of a homodimer partner receptor to induce the active receptor conformations. We recently demonstrated that three residues in the N-terminus of the RXFP1 LDLa module are potentially involved in hydrophobic interactions with the receptor to drive activation. RXFP2 shares two out of three of the residues implicated, suggesting that the two LDLa modules could be interchanged without adversely affecting activity. However, in 2007 it was shown that a chimera consisting of the RXFP1 receptor with its LDLa swapped for that of RXFP2 did not signal. We noticed this construct also contained the RXFP2 region linking the LDLa to the leucine-rich repeats. We therefore constructed chimeric RXFP1 and RXFP2 receptors with their LDLa modules swapped immediately C-terminally to the final cysteine residue of the module, retaining the native linker. In addition, we exchanged the TM domains of the chimeras to explore if matching the LDLa module with the TM domain of its native receptor altered activity. All of the chimeras were expressed at the surface of HEK293T cells with ligand binding profiles similar to the wild-type receptors. Importantly, as predicted, ligand binding was able to induce cAMP-based signaling. Chimeras of RXFP1 with the LDLa of RXFP2 demonstrated reduced H2 relaxin potency with the pairing of the RXFP2 TM with the RXFP2 LDLa necessary for full ligand efficacy. In contrast the ligand-mediated potencies and efficacies on the RXFP2 chimeras were similar suggesting the RXFP1 LDLa module has similar efficacy on the RXFP2 TM domain. Our studies demonstrate the LDLa modules of RXFP1 and RXFP2 modulate receptor activation via a similar mechanism.

## Introduction

Relaxin family peptide receptor (RXFP) 1 and RXFP2 are class A G-protein coupled receptors (GPCR). They are therefore members of the largest gene family in humans (1), and GPCRs are currently the target of more directed drugs than any other gene family (2). While RXFP1 and RXFP2 both possess the representative 7 transmembrane (TM) spanning α-helices, they also contain a large extracellular domain, consisting of 10 leucine-rich repeats (LRR) tethered to a single low-density lipoprotein type A (LDLa) module (3) by an uncharacterized linker. The LRRs are the primary binding site for the cognate ligands, relaxin (RXFP1) and insulin-like peptide 3 (INSL3) (RXFP2), while the LDLa module is essential for signaling (4). As the receptors have been shown to form constitutive homodimers (5, 6) it is hypothesized that the LDLa module acts as a secondary ligand, possibly interacting with the extracellular loops of the TM region of a homodimer partner receptor to induce the conformational change necessary for signaling (7). RXFP1 and RXFP2 are the only known mammalian GPCRs to contain an LDLa module and thus the potential role of this module in signal activation is unique. The module itself requires the formation of three disulfide bonds between six conserved cysteine residues, as well as the presence of a single bound calcium ion to maintain its active, globular structure (8). Replacing the native LDLa module of RXFP1 with the structurally similar but functionally distinct LB2 module from the low-density lipoprotein receptor (LDLR) gives rise to a chimera that can bind ligand like the wild-type (WT), but cannot signal (8, 9). The RXFP1-LB2 chimera was used to identify key residues needed for signaling in a gain-of function study complemented with equivalent loss-of-function mutations and a detailed structural analysis (9). In this way a prospective binding surface was identified involving Leu7, Tyr9, and Lys17, which are proposed to contribute to the activation of RXFP1 using hydrophobic contacts.

**Figure 1:**
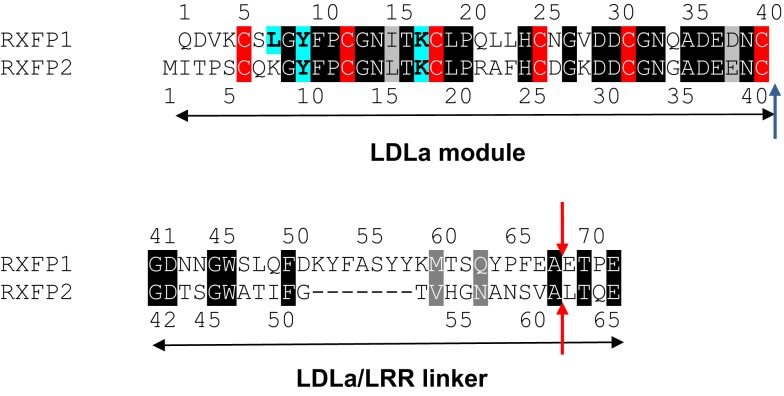
**Alignment of the RXFP1 and RXFP2 LDLa module and linker sequences**. Identical residues are boxed in black and similar residues are boxed in gray while the conserved cysteine residues are boxed in red. The residues recently identified as being involved in RXFP1 ligand-mediated activation (9) are highlighted in blue in both RXFP1 and where they are conserved in RXFP2. A blue arrow shows where our swapped modules are cleaved. Red arrows show the point of cleavage for the swapped modules in the chimera made by Kern et al. (12).

A similar mechanism of action of the LDLa module by the two receptors is implied by the degree of sequence similarity they share. The LDLa modules of RXFP1 and RXFP2 share 60% sequence similarity (Figure 1), each possessing features common to other LDLa modules characterized from the LDLR including the six cysteines that form disulfide bonds, and the motif AspxxxAspxxAspxxAspGlu (where x is any residue) that binds a calcium ion. Many LDLa modules characterized from the LDLR utilize this motif to not only ligate the calcium ion but contribute to protein–protein interactions (10), while residues important to RXFP1 function have been mapped to the N-terminal region of the LDLa (9). Two residues key to the function of RXFP1, Tyr9, and Lys17, are conserved in the RXFP2 LDLa module and it is reasonable to assume they function in a similar manner to induce signal activation. Importantly, it has been shown that chimeras, named RXFP1/2 and RXFP2/1, in which the entire ectodomain of the receptors are swapped are still able to signal, albeit with lowered activity compared to WT (11). This implies that the LDLa module of RXFP2 can compensate for the module of RXFP1 and vice versa. It would seem reasonable to assume then that simply swapping the LDLa modules between RXFP1 and RXFP2 would also yield chimeras capable of signaling. However, in a study by Kern and colleagues in 2007 where they replaced the RXFP1 LDLa module with that of RXFP2, they reported that the chimera did not signal, concluding that the cAMP signaling function of RXFP1 was only possible with its native LDLa module (12).

**Figure 2:**
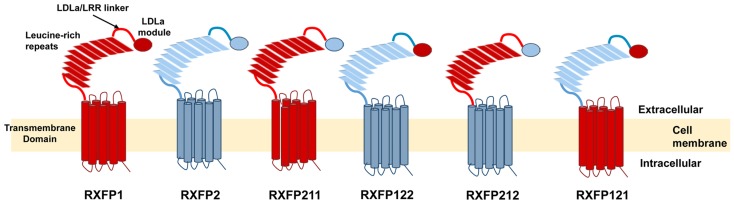
**Schematic representation of the RXFP1 and RXFP2 receptors compared to the RXFP211, RXFP122, RXFP212, and RXFP121 chimeric receptors**. The domains of receptors are labeled on the RXFP1 receptor. RXFP1 domains are in blue while RXFP2 domains are in red.

The present study aims to further investigate this seeming contradiction, and establish why an ectodomain-swapped chimera of RXFP1 with RXFP2 can signal, while an equivalent LDLa-swapped chimera cannot. Upon scrutiny of the constructs made by Kern et al., it would appear that the point chosen to swap the LDLa modules was 27 residues C-terminally to the final cysteine (Cys40) necessary for LDLa structural integrity for RXFP1. Importantly, the equivalent region of RXFP2 which was replaced in the chimera is seven amino acids shorter (Figure 1), meaning that the resulting chimera had a shorter stretch of amino acids linking the LDLa module to the LRRs than WT RXFP1. We therefore designed chimeric constructs of both RXFP1 and RXFP2 that had their LDLa modules swapped immediately C-terminally to the aforementioned cysteine similar to our RXFP1-LB2 chimera (8), meaning that only the LDLa module itself was swapped, and none of the adjoining linker residues. We call these constructs RXFP211 and RXFP122 (Figure 2) based on the nomenclature utilized previously for ectodomain-swapped RXFP1 and RXFP2 receptors (11). As further investigation into the possibility of a receptor-specific interaction between the LDLa module and the TM domain, we also matched the LDLa module with the complementary TM domain, to establish if an improvement in signaling activity would be observed. These constructs are named RXFP212 and RXFP121 (Figure 2). The resulting chimeras were tested for surface expression, binding of ligand, and cAMP-based signaling.

## Materials and Methods

### Hormones and cell lines

Recombinant human gene-2 relaxin (H2 relaxin) peptide was kindly provided by Corthera and human INSL3 was chemically synthesized as previously described (13). Human embryonic kidney (HEK) 293T cells (ATCC #CRL-1573; American Type Tissue Culture Collection) used to express receptors were maintained in Dulbeccos’ modified eagle medium (DMEM) supplemented with 10% fetal bovine serum (FBS), 1% l-glutamine and 1% penicillin/streptomycin (referred to as complete DMEM) in incubators (Thermo scientific) maintained at 37°C, with 5% CO_2_ and 85% humidity.

### Receptor constructs

RXFP1, RXFP2, and chimeric constructs were cloned into a pcDNA3.1™/Zeo+ AmpR mammalian expression vector (Invitrogen, Carlsbad, CA, USA) which contained an N-terminal FLAG tag and a bovine prolactin signal sequence (3) and has been shown not to interfere with receptor activity (14). The chimeric receptor constructs were made using overlap PCR. The LDLa modules were first generated using the relevant LDLa module as template and PCR primers that would anneal to the two ends of the LDLa module (Table 1). The amplification PCR mixture contained 100 ng of DNA template, 10 μl of 10× PCR buffer, 1 μl of each primer, 0.5 μl of dNTP mix, 2 μl of MgCl_2_, 0.5 μl of Taq (Bioline Velocity) and distilled, de-ionized water to make up to a total of 50 μl. PCR conditions were 2 min at 98°C, then 30 cycles of 98°C (30 s), 58°C (30 s), 72°C (15 s) followed by a 7 min extension at 72°C. The LRR/TM regions of each construct were similarly amplified (Table 1) and the two sections of DNA were annealed together in a PCR reaction consisting of 200 ng of LDLa DNA, 50 ng of LRR/TM DNA, 10 μl of 5× PCR buffer, 1 μl of dNTP mix, 0.25 μl of Taq polymerase (Bioline Velocity), and made up to 50 μl with distilled, de-ionized water. The conditions used were 98°C (2 min) then 10 cycles of 98°C (30 s), 50°C (25 min), 72°C (5 min). The final annealed product was then amplified using the same protocol as in the other amplification steps, except that the 72°C step was carried out for 30 s instead of 15 s. Primers used were the forward (sense) primer used to amplify the LDLa sequence and the reverse (antisense) primer used to amplify the LRR/TM sequence for each construct (Table 1). The DNA was then digested with *Bam*HI and *Xho*I to create sticky ends that would correspond to sites in fresh, verified, and digested pcDNA3.1 Zeo+ vector. Ligation was carried out using T4 DNA ligase (Promega) at 4°C overnight or at room temperature for 3 h. The vectors were then transformed into DH5α *E. coli* cells using the heat shock method and resultant colonies were picked and grown before isolating the plasmid DNA using a PureLink^®^ Quick Plasmid Miniprep Kit. The entire insert of successful clones were sequenced to ensure the desired end product was correct, with no unintentional mutations incorporated.

**Table 1:** **Primers used in overlap cloning**.

Construct component	Template DNA	Primer (5′–3′DNA sequence)
RXFP2 LDLa	RXFP2 LDLa module	Sense: CATCATGGATCCGCCACCATGGACAGCAAAG
		Antisense: CAGAGACCATCCATTGTTGTCTCCACAGTTCTCTTCGTCCGCCCC
RXFP1 LDLa	RXFP1 LDLa module	Sense: CATCATGGATCCGCCACCATGGACAGCAAAG
		Antisense: CGCCCATCCACTAGTGTCACCACAGTTGTCCTCATCGGCCTG
RXFP211 LRR and TM	RXFP1	Sense: GGGGCGGACGAAGAGAACTGTGGAGACAACAATGGATGGTCTCTG
		Antisense: GAGAGCTCGAGTCATGAATAGGAATTGAGTCTCGTTG
RXFP212 LRR and TM	RXFP1/2	Sense: GGGGCGGACGAAGAGAACTGTGGAGACAACAATGGATGGTCTCTG
		Antisense: CATCATCATCTCGAGCTAGGAAACTGGTTTCATTATACTGTC
RXFP122 LRR and TM	RXFP2	Sense: CAGGCCGATGAGGACAACTGTGGTGACACTAGTGGATGGGCG
		Antisense: CATCATCATCTCGAGCTAGGAAACTGGTTTCATTATACTGTC
RXFP121 LRR and TM	RXFP2/1	Sense: CAGGCCGATGAGGACAACTGTGGTGACACTAGTGGATGGGCG
		Antisense: GAGAGCTCGAGTCATGAATAGGAATTGAGTCTCGTTG

### Receptor expression on HEK 293T cells

Transient transfections were performed using lipofectAMINE™ 2000 (Invitrogen) according to the manufacturer’s instructions. For competition binding assays chimeric receptors were selected for semi-stable expression and compared with cells stably expressing RXFP1 or RXFP2 (9). Semi-stable expression was achieved by selection of cells with Zeocin after transient transfection followed by fluorescence activated cell sorting (FACS) using a fluorescently tagged FLAG antibody 1 week later. For FACS analysis, cells were seeded in 10 cm dishes at 3 × 10^6^ cells, and grown overnight. The following day the cells were washed and resuspended in 1% FBS in phosphate buffered saline (PBS) (Gibco) (FBS/PBS) medium. Alexa 647 labeled anti-FLAG antibody (Invitrogen) was added at a 1:1000 dilution in FBS/PBS and cells were incubated on ice for 30 min, then passed through a filter and sorted using FACS (Becton Dickinson FACS Aria III). Only the top 10% of cells with fluorescence levels significantly higher than the background fluorescence in non-transfected HEK293T control cells were collected. The sorted cells were grown in complete DMEM in the presence of 200 μg/ml Zeocin until confluent. HEK293T cells stably expressing WT RXFP1 were used as a positive control in the FACS sort.

### Cell surface expression assays

The presence of the chimeric receptors at the surface of cells was assessed in triplicate, exploiting the FLAG epitope on their N-termini using the method described previously (15). Briefly, cells were transfected with receptor of interest for 24 h. Next, cells were fixed with 3.7% formaldehyde solution (Sigma-Aldrich), blocked with bovine serum albumin (BSA) and incubated with an anti-FLAG mouse monoclonal primary antibody (Sigma-Aldrich). Goat anti-mouse Alexa Fluor 488 was used as secondary antibody (Invitrogen). Finally, cells were lysed and transferred into black-walled 96-well plates for measurement using a BMG plate reader (Omega) at 495 nm excitation and 519 nm emission wavelengths. Data were analyzed using GraphPad PRISM (Graphpad Software, Inc.) and presented as mean percentage of WT RXFP1 or RXFP2 expression ± SEM of at least three independent experiments. Significance of receptor expression above background and compared to WT was assessed using one-way ANOVA and uncorrected Fisher’s LSD multiple comparison test.

### Ligand binding assays

Competition binding assays were performed on whole cells as described previously, using Europium labeled INSL3 (Eu-INSL3) (16) and Europium labeled H2 relaxin (Eu-H2 relaxin) (17). Increasing concentrations of unlabeled ligand were used in conjunction with labeled equivalents, and non-specific binding was established using 1 μM unlabeled ligand. Readings were taken in triplicate and read on a BMG plate reader (Omega) in clear-bottomed, opaque-walled 96-well plates (PerkinElmer). Data were analyzed using GraphPad PRISM and presented as mean percentage specific binding ± SEM of at least three independent experiments. A non-linear regression one-site binding curve was then fitted and resulting pIC_50_ values were subjected to one-way ANOVA and uncorrected Fisher’s LSD comparison test.

### cAMP activity assays

Cells were assayed for cAMP signaling by co-transfection of receptors with a pCRE β-galactosidase (β-gal) reporter construct as previously described (18). Cells were stimulated for 6 h at 37°C with H2 relaxin or INSL3 at dilutions varying from 1 μM to 0.001 nM. Pooled data from at least three separate experiments, each performed in triplicate were presented as percentages of the maximum response induced by 5 μM Forskolin ± SEM. A non-linear regression sigmoidal dose-response curve was then fitted using GraphPad PRISM and resulting pEC_50_ and maximum response (*E*_max_) values were subjected to one-way ANOVA and uncorrected Fisher’s LSD comparison test.

## Results

### Characterization of RXFP211 and RXFP212

**Figure 3:**
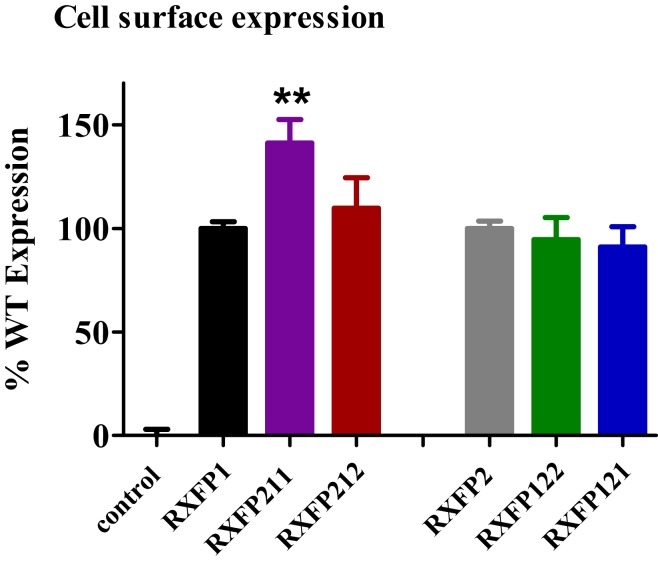
**Cell surface expression of chimeric receptors compared to the RXFP1 and RXFP2 wild-type (WT) receptors**. Data are expressed as mean ± SEM of triplicate determinations from at least three independent experiments. **p < 0.01 compared to RXFP1.

Cell surface expression assays on the chimeric constructs showed that all receptors were expressed at the cell membrane, since their fluorescence profiles were consistently equal to or greater than that displayed by the WT receptors (Figure 3). Both RXFP211 and RXFP212 were expressed more highly than WT RXFP1, which is consistent with findings by Kern et al. for their LDLa-swapped chimera (12). However, only RXFP211 showed significantly (*p* < 0.01) higher expression than RXFP1 (141.2 ± 11.3% of RXFP1 expression) (Figure 3; Table 2).

**Table 2:** **Pooled binding affinity (pIC_50_), cell surface expression and cAMP activity (pEC_50_ and *E*_max_) data for chimeric receptors in comparison to RXFP1 and RXFP2**.

Receptor	Cell surface expression	Eu-H2 relaxin competition binding (pIC_50_)	Eu-INSL3 competition binding (pIC_50_)	INSL3 stimulation		H2 relaxin stimulation	
				pEC50	*E*_max_ (%Forskolin)	pEC50	*E*_max_ (%Forskolin)
	**% RXFP1**
RXFP1	100 ± 3.23 (8)	8.77 ± 0.10 (7)	ND	ND	ND	10.89 ± 0.07 (4)	102.4 ± 3.3 (3)
RXFP211	141.2 ± 11.3 (7)**	8.64 ± 0.15 (8)	ND	ND	ND	10.28 ± 0.15 (3)	64.5 ± 3.4 (3)*
RXFP212	109.7 ± 14.7 (5)	8.43 ± 0.17 (4)	ND	ND	ND	9.19 ± 0.03 (3)**	99.9 ± 5.3 (3)
	**% RXFP2**
RXFP2	100 ± 3.5 (4)	ND	8.79 ± 0.06 (6)	10.26 ± 0.42 (3)	115.1 ± 9.2 (3)	9.13 ± 0.06 (3)	102.6 ± 16.5 (3)
RXFP122	94.5 ± 10.7 (4)	ND	8.35 ± 0.08 (6)^##^	9.67 ± 0.20 (5)	93.9 ± 3.6 (5)	7.95 ± 0.10 (6)^##^	109.8 ± 5.0 (6)
RXFP121	91.0 ± 9.8 (5)	ND	8.43 ± 0.17 (3)^#^	10.40 ± 0.34 (5)	93.7 ± 14.6 (5)	8.22 ± 0.17 (4)^#^	101.4 ± 8.8 (4)

*p < 0.05; **p < 0.01 vs. RXFP1; ^#^*p* < 0.05; ^##^*p* < 0.01 vs. RXFP2; ND – not determined.

**Figure 4:**
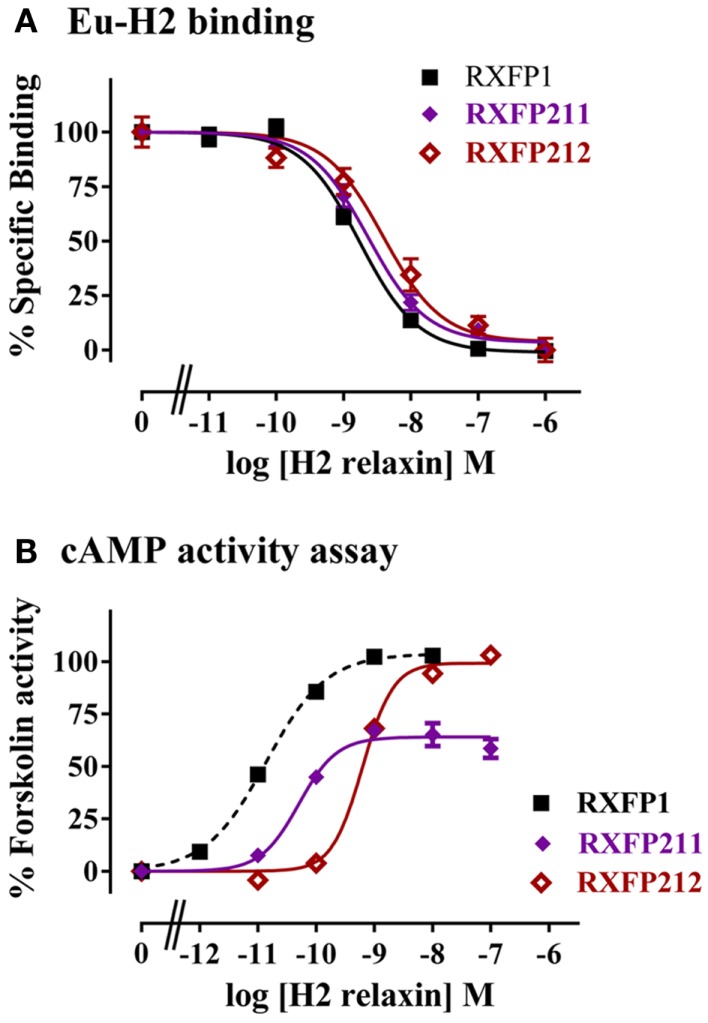
**Activity of RXFP1 chimeric receptors compared to RXFP1**. **(A)** Competition binding using Eu-labeled H2 relaxin. **(B)** H2 relaxin-induced cAMP responses. cAMP activity is expressed as the percentage of the 5 μM Forskolin-stimulated response for each receptor. Note the data for the RXFP211 receptor has been normalized for cell surface expression (see text for details). Data are expressed as mean ± SEM of triplicate determinations from at least three independent experiments.

The construct RXFP211 is made up of the LDLa module from RXFP2 and the LRRs and TM domain of RXFP1. RXFP212 contains the same domains except that the TM domain is from RXFP2 (Figure 2). As high affinity ligand binding is driven by the LRRs, binding was assessed in comparison to RXFP1 using a competition binding assay with Eu-H2 relaxin. As expected binding was unaltered in comparison to WT as the LRR sequence was equivalent to the native receptor in each of the constructs (Figure 4A).

The two LDLa-swapped receptors were tested for cAMP production using a reporter gene assay. As the RXFP211 chimera had significantly different cell surface expression to RXFP1 the results have been normalized to cell surface expression for this construct only. While both chimeras were able to induce cAMP activity in response to H2 relaxin, the potency was reduced, with the pEC_50_ of H2 relaxin on RXFP211 being 10.28 ± 0.15, and that of H2 relaxin on RXFP212 9.19 ± 0.03, the latter of which is significantly different (*p* < 0.01) from WT RXFP1 (pEC_50_ 10.89 ± 0.03) (Figure 4B; Table 2). Importantly the efficacy of H2 relaxin-induced cAMP responses at RXFP211 was significantly reduced compared to RXFP1 with a maximum Forskolin response of 64.02 ± 3.4% compared with 102.4 ± 3.3% (*p* < 0.05). The replacement of the RXFP1 TM domain with that of RXFP2 in RXFP212 restored the H2 relaxin stimulated efficacy to 99.9 ± 5.3% Forskolin response (Figure 4B; Table 2).

### Characterization of RXFP122 and RXFP121

**Figure 5:**
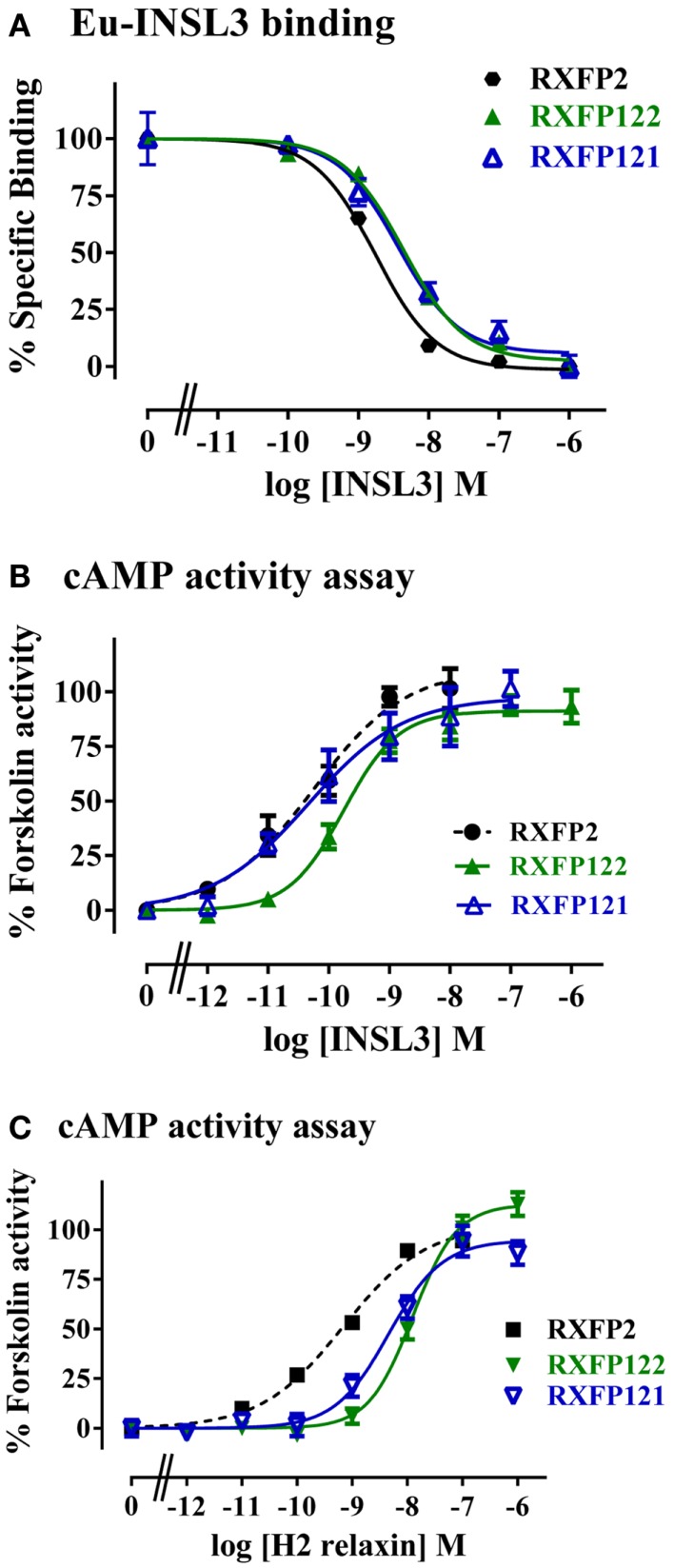
**Activity of RXFP2 chimeric receptors compared to RXFP2**. **(A)** Competition binding using Eu-labeled INSL3. **(B)** INSL3-induced cAMP responses. **(C)** H2 relaxin-induced cAMP responses. cAMP activity is expressed as the percentage of the 5 μM Forskolin-stimulated response for each receptor. Data are expressed as mean ± SEM of triplicate determinations from at least three independent experiments.

RXFP122 has the TM and LRR domains from RXFP2 and the LDLa module from RXFP1. RXFP121 has the LDLa module and the TM region from RXFP1 and the LRRs from RXFP2 (Figure 2). Cell surface expression for these two constructs was not significantly different from WT RXFP2 levels (Figure 3; Table 2). Binding assays also showed similar patterns of binding to RXFP2, although pIC_50_ values for INSL3 binding were found to be significantly different from RXFP2 (*p* < 0.05 for RXFP121 and *p* < 0.01 for RXFP122) (Figure 5A; Table 2). Given that the constructs had the LRR region from RXFP2, we tested them for signaling with both INSL3 and H2 relaxin, as these ligands are both known to activate RXFP2 (3). While the pEC_50_ values were not significantly different from RXFP2 on INSL3 stimulation, they were reduced from the 9.13 ± 0.06 seen in RXFP2 in response to H2 relaxin to 7.95 ± 0.10 for RXFP122 (*p* < 0.01) and 8.22 ± 0.17 for RXFP121 (*p* < 0.05) (Figures 5B,C; Table 2). Importantly, ligand-mediated maximum responses (*E*_max_) did not differ significantly from RXFP2.

## Discussion

The activation mechanisms of RXFP1 and RXFP2 represent a unique paradigm in GPCR functioning, since these are the only two mammalian GPCRs that possess an LDLa module. When found in the LDLR and related receptors, these modules are typically involved in protein–protein interactions, and are thus involved in a variety of interactions both with peptides and other molecules (19). The evidence suggests that the LDLa modules on the RXFP receptors are also involved in protein interaction, although rather than being involved in ligand binding, we have hypothesized that the LDLa module binds with another domain on the receptor, and that this interaction is necessary to induce the conformational changes needed for signaling to occur (7). Nevertheless, while in most LDLR family receptors the binding surface of the LDLa modules seems to involve C-terminal residues, which are also involved in Ca^2+^ binding (10, 20), the RXFP1 LDLa module appears to have a binding surface that involves residues in the N-terminal region, and the hydrophobic regions of the side-chains of Leu7, Tyr9, and Lys17 have been implicated in mutational investigations on RXFP1 (9). Both Tyr9 and Lys17 are conserved between RXFP1 and RXFP2 (Figure 1), and although they may interact differently in each case, their presence implies a similar mechanism of activation in the two receptors, also suggesting the LDLa modules of each receptor could be swapped with limited effect on receptor activation. It was therefore surprising that it was reported that when the LDLa module of RXFP2 was swapped into RXFP1 that the chimeric receptor was inactive (12). However upon investigation of the chimeric receptor design we noted that the authors had designed the chimeric construct with additional amino acid residues from the RXFP2 linker region C-terminal of the LDLa module. Importantly this region of RXFP2 is shorter than that in RXFP1 and therefore when the chimera was produced the linker between the LRRs and the LDLa of the chimeric RXFP1 receptor was seven amino acids shorter. In this study we therefore first designed a chimeric RXFP1/RXFP2 receptor construct whereby the RXFP2 LDLa module was swapped at the C-terminal cysteine residue of the module. By reducing the swapped domain to its functional minimum length and size, we were able to produce a functional RXFP211 receptor as hypothesized, although the efficacy of signaling was reduced compared to the native receptor. Hence it is likely that the inactivity of the Kern chimeric RXFP1 construct was due to the shortening of the linker region between the LRRs and LDLa rather than the swapping of the LDLa modules.

While Kern et al. only made the RXFP1 chimera with the LDLa module of RXFP2 attached, we sought to characterize both this and the equivalent RXFP2 construct with the LDLa from RXFP1. We hypothesized that such chimeras would be informative in relation to common and distinct mechanisms of activation by the LDLa module. Additionally, we explored the concept that the LDLa is exerting its effect by interacting with the TM domain of the receptor in a specific manner by creating LDLa chimeras with matched TM domains in RXFP121 and RXFP212. The chimeric receptors were all expressed at the cell surface indicating they were folded correctly and able to be trafficked to the cell surface. The RXFP211 chimera had significantly higher cell surface expression as previously demonstrated for the chimera produced by Kern et al. (12). Binding affinities of both RXFP1 and RXFP2 chimeras were slightly lower than the WT receptors with only the RXFP2 chimeras being statistically different. These differences are likely due to the lower expression levels of the receptors in the cells used for ligand binding. The sensitivity of the binding assays requires stable or semi-stable cell lines and the expression levels of the RXFP1 and RXFP2 stables were considerably higher as they were high expressing monoclonal stable lines (data not shown). These lower expression levels would influence the pIC_50_ values and unfortunately there was not sufficient Eu-labeled ligand to perform saturation binding to obtain *K*_d_ values. However, we have previously demonstrated that RXFP1 and RXFP2 receptors lacking the LDLa module bind ligand normally (4). Hence the slight reductions in pIC_50_ values are unlikely to be related to the LDLa module swaps.

As anticipated the chimeric receptors did show differences in ligand-mediated cAMP activity compared to the WT receptors. This was most obvious for the RXFP1 chimeras where H2 relaxin demonstrated both decreased potency as well as decreased % maximum Forskolin activity on RXFP211. The shift in pEC_50_ was similar to what we have previously demonstrated when the key RXFP1 LDLa residue Leu7 is mutated to Lysine as it is in the RXFP2 LDLa module (9). The decreased *E*_max_ values may reflect the mismatch of the LDLa with the TM domain resulting in the RXFP2 LDLa module acting like a partial agonist on the RXFP1 receptor. Notably, we have seen similar decreases in *E*_max_ with multiple mutations of the RXFP1 LDLa module (8, 9). Furthermore, H2 relaxin activation of RXFP1-LB2 gain-of-function receptors also resulted in lower *E*_max_ values compared to RXFP1 (9). Interestingly the replacement of the TM domains with that from RXFP2 in the chimera RXFP212 resulted in full H2 relaxin stimulated efficacy but coupled with lower potency. Hence the RXFP2 LDLa module is now acting more like a full agonist as potentially the matching of the LDLa module and the TM domain allows for optimal interactions to increase the probability of the receptor becoming fully activated. The decreased ligand potency on RXFP212 may be due to subtle differences in the LRRs that confer decreased coupling efficiency between ligand binding and LDLa mediated activation. Similarly, because RXFP1 and 2 are multi-domain receptors, it is these subtle mismatches (in LRRs and TM domains) that lead to the changes in ligand potency seen in all the chimeric receptors. Our previous studies with RXFP2 LRR chimeric receptors have highlighted that changes in the ectodomain structure have profound effects on ligand-mediated activation (21). Furthermore, as indicated above in the gain-of-function analysis using the RXFP1-LB2 construct, full restoration of signaling was not obtained, despite inclusion of sequence that corresponded to the three-dimensional structure of the interactive surface of the RXFP1 LDLa module (9). In contrast the RXFP2 chimeric receptors had more modest effects on receptor activity with only a slight decrease in the potency of H2 relaxin at both chimeras which may reflect the different mechanism by which H2 relaxin binds and activates RXFP2 (21, 22). The similar activity of the two chimeras is in contrast to the RXFP1 chimeras and may reflect the fact that H2 relaxin is a ligand of both receptors and the RXFP2–INSL3 interaction evolved more recently (23, 24). Hence the RXFP1 LDLa module may have equal efficacy on the RXFP2 TM domains. This hypothesis is supported by the similar efficacy of ligands on the previously reported RXFP1/2 chimera (11) which matches an RXFP1 LDLa with the RXFP2 TM domain in addition to the reduced ligand efficacy on the RXFP2/1 chimera in this same study (11) again highlighting a partial agonist effect of the RXFP2 LDLa on the RXFP1 TM domain.

It should also be pointed out that it is highly unlikely that changes in potency or efficacy in the receptor chimeras is related to decreased efficiency of homodimerization of the receptors. Our previous studies on dimerization of RXFP1 (6) and RXFP2 (5) have demonstrated that homodimerization is primarily driven by TM interactions with some potential stabilizing influence of the ectodomains. As the TM and LRR regions would be matched in homodimers of the chimeric receptors and there was no evidence of profound changes in cell surface expression or efficacy changes on our chimeras or with RXFP1/2 and RXFP2/1 previously (11), it is therefore likely that the chimeric receptors are constitutive homodimers like the WT receptors.

Taken together, this study has shown that the LDLa modules of RXFP1 and RXFP2, which are unique among GPCRs, behave in a comparable fashion to one another, and that their mechanism of action must therefore be closely related. Additionally it is clear that the length of the linker region between the LRRs and the LDLa module is important for RXFP1 function. This information can be used to further elucidate a model of activation, as we gradually clarify the myriad of different elements that come into play upon binding and activation of these complex receptors. Given that relaxin has been implicated in various pathologies including cancer (25) and that it is has recently successfully completed a phase 3 clinical trial for the treatment of chronic heart failure (26) any knowledge of the activation of its cognate receptor and family members leads us closer to the development of small molecule analogs and antagonists that may be therapeutically relevant.

## Conflict of Interest Statement

The authors declare that the research was conducted in the absence of any commercial or financial relationships that could be construed as a potential conflict of interest.
